# Effects of Fetal Images Produced in Virtual Reality on Maternal-Fetal Attachment: Randomized Controlled Trial

**DOI:** 10.2196/43634

**Published:** 2023-02-24

**Authors:** Kyong-No Lee, Hyeon Ji Kim, Kiroong Choe, Aeri Cho, Bohyoung Kim, Jinwook Seo, Woojae Myung, Jee Yoon Park, Kyung Joon Oh

**Affiliations:** 1 Department of Obstetrics and Gynecology Seoul National University Bundang Hospital Seoul National University College of Medicine Seongnam-si, Gyeonggi-do Republic of Korea; 2 Department of Computer Science and Engineering Seoul National University Seoul Republic of Korea; 3 Division of Biomedical Engineering Hankuk University of Foreign Studies Gyeonggi-do Republic of Korea; 4 Department of Neuropsychiatry Seoul National University Bundang Hospital Seoul National University College of Medicine Seongnam-si, Gyeonggi-do Republic of Korea

**Keywords:** maternal-fetal attachment, virtual reality, ultrasound, pregnancy, fetus, postpartum depression, pediatric, mobile app, mental well-being, mobile health app, maternal health, women's health

## Abstract

**Background:**

Maternal-fetal attachment (MFA) has been reported to be associated with the postpartum mother-infant relationship. Seeing the fetus through ultrasound might influence MFA, and the effect could be increased by more realistic images, such as those generated in virtual reality (VR).

**Objective:**

The aim was to determine the effect of fetal images generated in VR on MFA and depressive symptoms through a prenatal-coaching mobile app.

**Methods:**

This 2-arm parallel randomized controlled trial involved a total of 80 pregnant women. Eligible women were randomly assigned to either a mobile app–only group (n=40) or an app plus VR group (n=40). The VR group experienced their own baby’s images generated in VR based on images obtained from fetal ultrasonography. The prenatal-coaching mobile app recommended health behavior for the pregnant women according to gestational age, provided feedback on entered data for maternal weight, blood pressure, and glucose levels, and included a private diary service for fetal ultrasound images. Both groups received the same app, but the VR group also viewed fetal images produced in VR; these images were stored in the app. All participants filled out questionnaires to assess MFA, depressive symptoms, and other basic medical information. The questionnaires were filled out again after the interventions.

**Results:**

Basic demographic data were comparable between the 2 groups. Most of the assessments showed comparable results for the 2 groups, but the mean score to assess interaction with the fetus was significantly higher for the VR group than the control group (0.4 vs 0.1, *P*=.004). The proportion of participants with an increased score for this category after the intervention was significantly higher in the VR group than the control group (43% vs 13%, *P*=.005). The feedback questionnaire revealed that scores for the degree of perception of fetal appearance all increased after the intervention in the VR group.

**Conclusions:**

The use of a mobile app with fetal images in VR significantly increased maternal interaction with the fetus.

**Trial Registration:**

ClinicalTrials.gov NCT04942197; https://clinicaltrials.gov/ct2/show/NCT04942197

## Introduction

Pregnancy brings various lifelong psychological changes and frequently affects a woman’s values, identity, marital relationship, parenting, and mood [1-3]. Depression is a serious psychological complication of childbearing, and its prevalence has increased during the COVID-19 pandemic [4]. Prenatal check-ups include many laboratory tests and ultrasonographic examinations, usually reassuring pregnant women by confirming fetal well-being; however, this can also cause stress, and depression or anxiety can develop [2,5]. Postpartum depression is a critical problem, since it results in various harmful consequences, such as avoidance of childcare, child abuse, and suicide [6-9].

Several studies have revealed that strong maternal emotions and bonding with the baby have protective effects against postpartum depression and anxiety, and these studies have tried to determine which factors stimulate or improve maternal-fetal attachment (MFA) [10,11]. Delavari et al [12] demonstrated a significant inverse association between MFA and the development of postpartum depressive symptoms in a longitudinal study. Pregnant women with weak MFA are less likely to engage in health-promoting activities and more likely to be reluctant to perform childcare; thus, adverse outcomes in behavior and development of their children might increase [13-15]. In the natural course, MFA increases gradually as gestation progresses, stabilizes in late pregnancy, helps adaptation to physiological changes, and determines maternal responsibility [16,17]. Güney and Uçar [18] reported that maternal subjective recognition of fetal movement enhanced MFA.

Virtual reality (VR) is the newest trend in computer-based technology that simulates objects or situations regardless of location or time [19,20]. It has been applied in numerous fields of health care, such as providing treatment [21-23], facilitating pain management [24-26], simulating surgery [27,28], providing guidance for rehabilitation [29], and enhancing medical education [30,31]. Because pregnant women are not able to see their babies before giving birth, they perceive the existence of the fetus by indirect factors, such as fetal movement or grayscale videos viewed during ultrasonography examinations. Since VR, with the assistance of ultrasonography, can reproduce the appearance of the fetus very realistically, we hypothesized that the vivid images of the fetus possible with VR could help promote MFA.

The purposes of this study were to investigate whether fetal images generated in VR helped pregnant women to imagine or perceive fetal appearance and to determine the positive effect on MFA and the protective effect against depressive symptoms.

## Methods

### Study Design

This 2-arm, parallel randomized controlled trial was conducted at the Department of Obstetrics and Gynecology at Seoul National University Bundang Hospital, Republic of Korea. Participants were recruited among pregnant women who visited the institution for routine prenatal check-ups and pregnancies after 20 weeks of gestation and were identified as being eligible. Patients with a history of any psychiatric disorder (eg, mood disorder or anxiety disorder) were excluded from this study. Recruitment began in June 2021 and enrollment was completed in October 2021.

The target sample size was calculated based on changes in Cranley score reported in a previous study [32]. Given an average Cranley attachment score of 2.8 (SD 0.51), 32 patients were required in each study arm to determine an increase in Cranley score of 0.3 using the software G*Power 3, with an α value of .05, a power of 80% (β=.20), and a 2-tailed independent *t* test (or Student *t* test) [33]. Allowing for a 25% dropout rate, we decided to enroll a total of 88 patients (44 participants per arm).

### Ethics Approval

Prior to initiation of this study, it was approved by the Seoul National University Bundang Hospital Institutional Review Board (B-2106-688-302), and the protocol was registered on ClinicalTrials.gov (NCT04942197). Written informed consent was obtained from all participants.

### Randomization and Protocol

**Figure 1:**
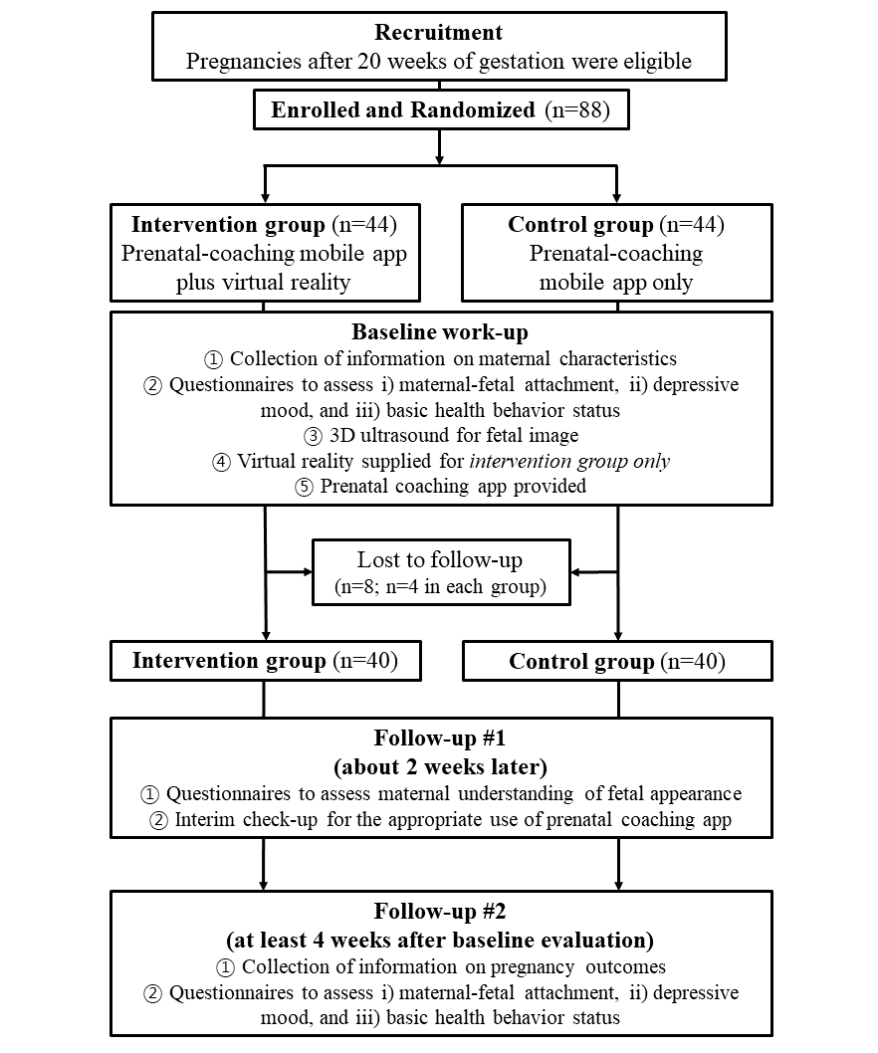
Flowchart of participation in the study.

During the study period, 88 eligible women were recruited and assigned randomly to either the VR intervention group or a standard-care group at a ratio of 1:1 by restricted randomization, which was generated with statistical software from the Medical Research Collaborating Center of the institution (Figure 1). Clinicians who met the participants in practice were not involved in the randomization process and were blinded to the allocation to each group until the participants completed the study protocol. Maternal baseline demographics and obstetric information were collected throughout the study period.

As the baseline work-up, the participants in both groups received and filled out three questionnaires: (1) an MFA assessment, (2) a depressive-symptoms assessment, and (3) a basic health behavior status and medical information survey. After they finished the 3 questionnaires, all participants underwent 3D fetal ultrasonography and were shown the fetal images on an ultrasonography monitor; however, only the VR intervention group was shown fetal images that merged ultrasonographic data to produce VR images; these were viewed in a headset. The fetal images were generated in VR from the data obtained through ultrasonography of the subject’s own fetus and were shown only to the VR group. A prenatal-coaching mobile app was provided afterwards, and consistent education on how to use the various functions of the app was given to all participants. Approximately 2 weeks later, the researchers checked the interim feedback on the proper use of the prenatal-coaching mobile app, and a fourth questionnaire to assess maternal understanding of fetal appearance was given to all participants. All participants completed the trial in 4 weeks, lasting from enrollment to the end of trial. They then repeated the 3 questionnaires from the beginning of the study (Figure 1). Every questionnaire was provided to the participants before and after fetal ultrasound examinations at the outpatient clinic; the average time for completion of each questionnaire was approximately 10 minutes.

**Figure 2:**
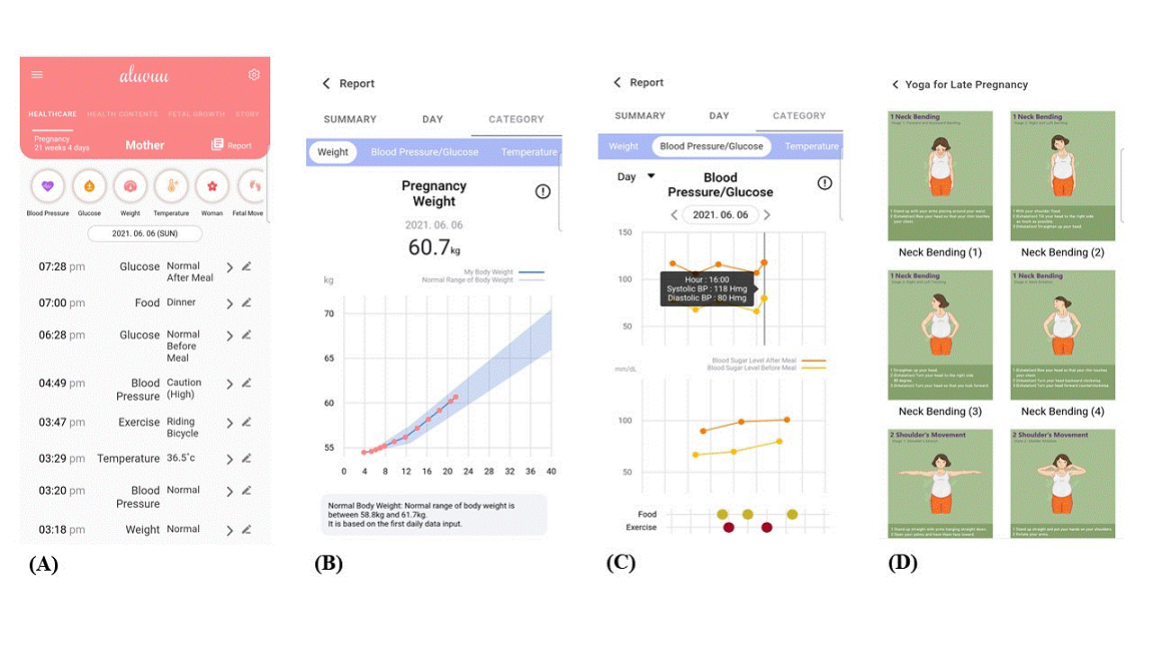
Screenshots of the prenatal-coaching mobile app: (A) glucose-concentration monitoring diary for mothers with gestational diabetes mellitus; (B) graph demonstrating maternal weight change; (C) diagram showing maternal blood pressure, glucose concentration, diet, and exercise at a glance; (D) information on helpful activities during pregnancy by gestational age (eg, yoga for late pregnancy).

All participants received the prenatal-coaching mobile app Aluvuu (available for both Android and iOS; Girjae Soft) when they were enrolled. This prenatal-coaching app provides services that enable submitting results for maternal weight and blood pressure and checking changes with intuitive graphs, and it includes a glucose-monitoring diary for participants with gestational diabetes mellitus or pregestational diabetes mellitus. Relevant information from authoritative guidelines on helpful activities (eg, yoga, exercise, and stretching) and a recommended diet were supplied according to gestational age (Figure 2).

**Figure 3:**
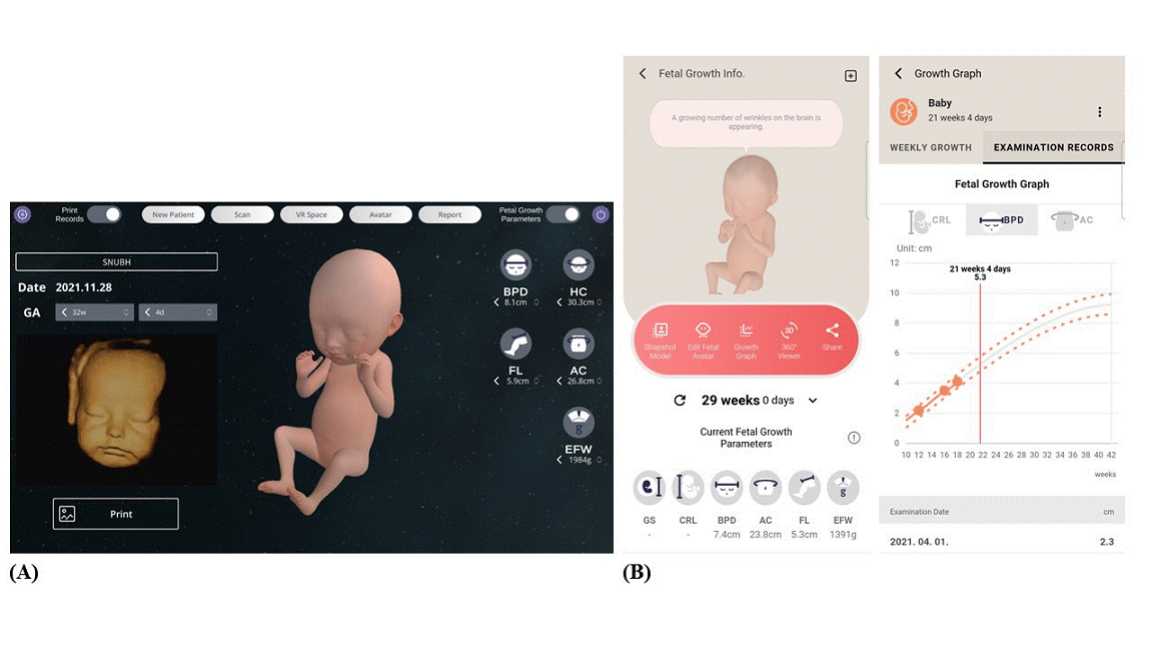
Fetal images produced in virtual reality: (A) fetal face and reconstructed image of the fetus generated in virtual reality based on 3D ultrasound–measured data of fetal body parts; (B) screenshots of the prenatal-coaching mobile app showing data converted from 3D ultrasounds and a graph demonstrating fetal growth (estimated based on data from ultrasound measurements).

**Figure 4:**
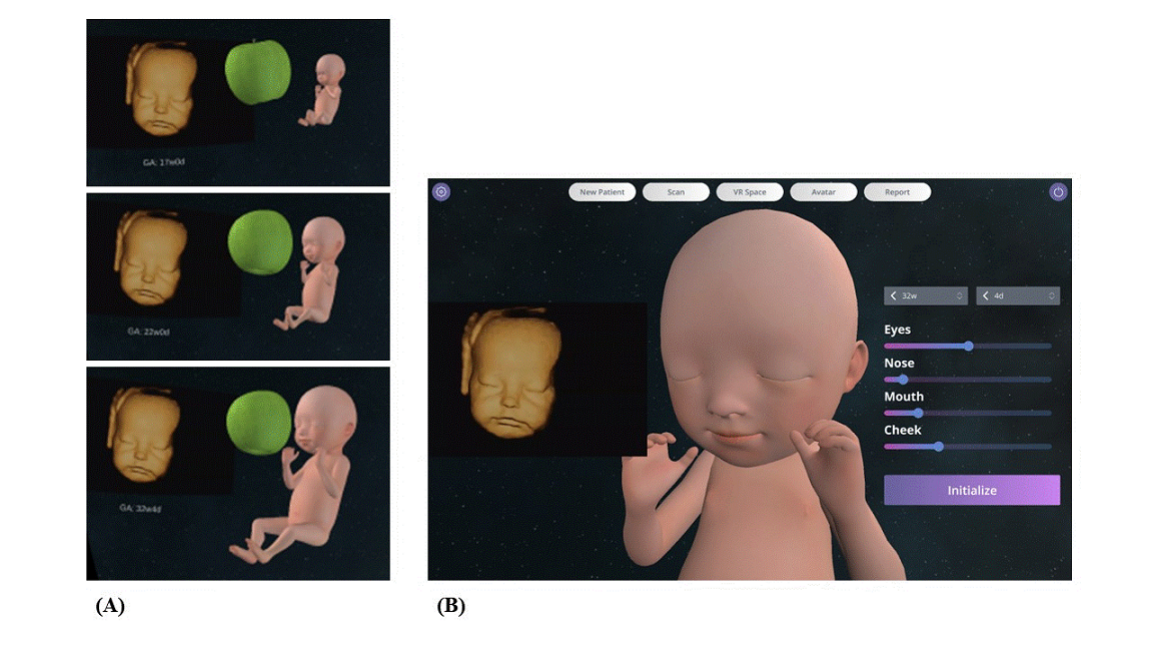
Additional services provided by the prenatal-coaching mobile app for the fetal images produced in virtual reality. (A) Progress in fetal growth at each gestational age in ultrasound images. (B) Interface for users to modify specific structures of the fetal face, such as the eyes, nose, mouth, and cheeks, in virtual reality.

All participants viewed 3D ultrasonography images; however, the VR experience and the fetal images produced in VR were provided only to the VR intervention group (Figure 3A). All the images generated in VR were sent to the prenatal-coaching mobile app, so that the users could see the images whenever they wanted. The users could share the saved pictures of their fetus with their family members and monitor growth via measurements of fetal body parts, such as head diameter (biparietal diameter), abdominal circumference, and leg length (femur length; Figure 3B). Progress in fetal growth at each ultrasonography examination was demonstrated in graphs that were saved in a private library folder. The users could compare the fetal image with everyday objects such as apples to help understand the actual size of the fetus (Figure 4A). In addition, the VR experience allowed the participants to actively engage their imagination about their expected baby by modifying specific structures of the fetal face, such as the eyes, nose, mouth, and cheeks, by touching the screen (Figure 4B).

### Questionnaires and Assessments

The most important questionnaire assessed MFA, which was the primary outcome of this study. Two well-known evaluation scoring systems were used: the Cranley method and the Condon method [34-36] (Multimedia Appendix 1; Table S1). The Cranley scoring method has 24 items that measure 5 behavior domains: *role taking*, *differentiation of self from fetus*, *interaction with the fetus*, *attributing characteristics to fetus*, and *giving of self* [37,38]. The choices for participants are scored from 1 to 5; thus, the total score ranges from 24 to 120. The Condon scale has 19 items that describe maternal attitude to the fetus; they are divided into 2 subscales [39]. The first subscale measures the quality of attachment and includes subjective maternal experiences, such as pleasure from interaction with the fetus and conceptualization of the fetus as an individual. The intensity of attachment is the second subscale; this measures the amount of time devoted to activities involving the fetus, such as talking, thinking, and dreaming about the fetus.

Maternal depressive symptoms were assessed by questionnaires that are generally used to screen for postpartum depression disorder, because there are no specific tests to determine pregnancy-related depressive symptoms that develop in the antenatal period. In fact, it is recommended that most postpartum depression scales be used regularly for high-risk patients starting with antenatal check-ups, not only during the postpartum period [40,41]. The Edinburgh Postnatal Depression Scale (EPDS) and Postpartum Depression Screening Scale (PDSS) were selected to evaluate the presence of depressive symptoms or disorder status for all participants [42,43]. The EPDS is the most widely applied screening test for postpartum depression; it has 10 items that rate the degrees of emotion that childbearing women have felt in the previous 7 days [44,45] The PDSS was developed to signify the concept or definition of postpartum depression; it uses 35 items to judge qualitative degrees of sleeping disturbance, eating disturbance, anxiety, insecurity, emotional lability, cognitive impairment, loss of self, guilt, shame, and contemplating harming oneself [46]. The recommended cutoff scores for minor postpartum depression are 60 for the PDSS and 9 for the EPDS [47].

The fourth questionnaire, which was given to participants at the interim follow-up, was a self-report to determine how much the participants understood the appearance of the fetus (Multimedia Appendix 1; Figure S1). The questions were as follows: (1) “Do you understand and imagine how big the actual size of your baby?” (2) “Do you understand and imagine the lengths of your baby’s arms and legs?” and (3) “Do you understand and imagine the detailed appearance of your baby’s face?” The participants could respond “definitely no,” “very little,” “moderately,” “very much,” or “definitely yes.” The replies were scored numerically from 1 to 5.

### Statistical Analysis

Maternal obstetric information and the scores from all the questionnaires were compared in the groups. Continuous variables were analyzed with a 2-tailed Student *t* test and proportions were compared using the Fisher exact test. A *P* value <.05 was considered significant. All statistical analyses were performed using SPSS (version 25.0; IBM Corp).

## Results

Among 88 participants enrolled at the beginning of the study, 4 from each group were lost to follow-up; therefore, data from 80 participants were included and analyzed. Maternal baseline characteristics, such as age, parity, height, weight, education, and use of assisted reproductive technology for the conception, were comparable between the 2 groups (Table 1). The proportion of twin pregnancies was 30% in the intervention group and 35% in the control group. Other obstetric complications included preeclampsia/superimposed preeclampsia, chronic hypertension, gestational diabetes mellitus, pregestational diabetes mellitus, preterm labor, preterm premature rupture of membranes, short cervical length, oligohydramnios, fetal growth restriction, alleged myoma uteri, and underlying malignancy; these were not statistically different between the 2 groups.

**Table 1:** Baseline demographics and clinical characteristics of the participants.

Characteristics		Intervention (n=40)	Control (n=40)	*P* value	
Age (years), n (%)		35 (3.9)	34 (3.3)	.95	
Nulliparous, n (%)		30 (75)	30 (75)	>.99	
Height (cm), mean (SD)		162.6 (5.7)	160.9 (4.2)	.14	
Weight at delivery (kg), mean (SD)		72.5 (9.6)	70.2 (10.5)	.34	
BMI at delivery (kg/m^2^), mean (SD)		27.3 (3.0)	27.1 (3.8)	.89	
Prepregnancy weight (kg), mean (SD)		58.9 (7.8)	57.1 (9.9)	.36	
Prepregnancy BMI (kg/m^2^), mean (SD)		22.3 (2.7)	22.0 (3.5)	.70	
**Education, n (%)**					.81
	High school graduate	3 (7.5)	1 (2.5)		
	Bachelor’s degree	28 (70)	33 (82.5)		
	Master’s degree or PhD	9 (22.5)	6 (15)		
Assisted reproductive technology, n (%)		15 (37.5)	21 (52.5)	.26	
Twins, n (%)		12 (30)	14 (35)	.81	
Preeclampsia/superimposed preeclampsia, n (%)		3 (7.5)	7 (17.5)	.31	
Chronic hypertension, n (%)		1 (2.5)	1 (2.5)	>.99	
Gestational diabetes, n (%)		4 (10)	7 (17.5)	.52	
Pregestational diabetes mellitus, n (%)		0 (0)	1 (2.5)	>.99	
Use of insulin due to diabetic disorder, n (%)		1 (2.5)	2 (5)	>.99	
High risk for preterm births, n (%)		8 (20)	7 (17.5)	>.99	
Preterm labor with use of tocolytics, n (%)		6 (15)	6 (15)	>.99	
Preterm premature rupture of membranes, n (%)		1 (2.5)	1 (2.5)	>.99	
Short cervical length in midtrimester, n (%)		6 (15)	2 (5)	.26	
Oligohydramnios, n (%)		2 (5)	1 (2.5)	>.99	
Fetal growth restriction, n (%)		1 (2.5)	1 (2.5)	>.99	
Alleged myoma uteri, n (%)		5 (12.5)	6 (15)	>.99	
Underlying malignancy, n (%)		1 (2.5)	1 (2.5)	>.99	

Table 2 demonstrates the primary outcome of this study. The mean values for gestational age at each evaluation, including the initial baseline evaluation and the follow-up evaluations, were comparable between the 2 groups. The median interval between the baseline evaluation and the second follow-up was 5.8 (IQR 4.6-7.9) weeks. The total scores obtained with the Cranley method and the Condon method did not show a statistical difference at either baseline or the follow-ups; however, among changes from baseline to follow-up, the mean value of 1 subscale of the Cranley test, *interaction with the fetus*, showed a greater increase in the intervention group than the control group (0.4, SD 0.5 vs 0.1, SD 0.4; *P*=.004). Table 3 shows the proportions of participants who had increased scores at follow-up evaluations compared to the initial baseline results. The rate of participants with an increased score was 43% for the intervention group, while it was 13% for the control group (*P*=.005).

**Table 2:** Comparison of maternal-fetal attachment in the intervention group and control group.

				Intervention (n=40), mean (SD)	Control (n=40), mean (SD)		*P* value
**Test results at initial baseline evaluation**							
	Gestational age at baseline evaluation (weeks)		27.8 (2.5)		28.2 (3.5)	.498	
	**Cranley test score**						
		Total	03.7 (9.0)		95.3 (10.5)	.48	
		*Role taking*	4.6 (0.6)		4.6 (0.7)	.72	
		*Differentiation of self from fetus*	3.9 (0.6)		3.8 (0.6)	.48	
		*Interaction with the fetus*	3.5 (0.7)		3.7 (0.6)	.18	
		*Attributing characteristics to fetus*	4.0 (0.5)		4.0 (0.6)	.69	
		*Giving of self*	4.0 (0.5)		4.2 (0.6)	.17	
	Total Condon test score		79.2 (8.6)		79.5 (8.6)	.89	
**Test results at follow-up evaluation**							
	Gestational age at follow-up evaluation (weeks)		33.3 (2.4)		33.9 (3.0)	.40	
	**Cranley test score**						
		Total	98.2 (8.6)		97.7 (8.2)	.79	
		*Role taking*	4.7 (0.6)		4.6 (0.6)	.45	
		*Differentiation of self from fetus*	3.7 (0.6)		3.8 (0.7)	.62	
		*Interaction with the fetus*	3.8 (0.6)		3.8 (0.6)	.85	
		*Attributing characteristics to fetus*	4.4 (0.5)		4.1 (0.5)	.06	
		*Giving of self*	4.2 (0.6)		4.3 (0.6)	.69	
	Total Condon test score		81.7 (7.3)		80.7 (7.3)	.52	
**Difference between initial evaluation and follow-up**							
	Interval from initial evaluation to follow-up (weeks)		6.3 (1.8)		5.9 (2.3)	.40	
	**Cranley test score**						
		Total	4.5 (7.3)		2.5 (7.1)	.21	
		*Role taking*	0.2 (0.5)		0.1 (0.6)	.39	
		*Differentiation of self from fetus*	0.0 (0.8)		0.1 (0.8)	.56	
		*Interaction with the fetus*	0.4 (0.5)		0.1 (0.4)	.004	
		*Attributing characteristics to fetus*	0.3 (0.7)		0.2 (0.6)	.36	
		*Giving of self*	0.1 (0.5)		0.1 (0.6)	>.9	
	Total Condon test score		2.5 (5.9)		1.2 (5.3)	.29	

**Table 3:** Proportions of participants with increased scores at follow-up compared to initial baseline results for maternal-fetal attachment evaluation.

Items		Intervention group (n=40), n (%)		Control group (n=40), n (%)		*P* value
**Cranley test score**						
	Total	28 (70)	26 (65)		.81	
	*Role taking*	8 (20)	7 (18)		>.99	
	*Differentiation of self from fetus*	9 (23)	9 (23)		>.99	
	*Interaction with the fetus*	17 (43)	5 (13)		.005	
	*Attributing characteristics to fetus*	15 (38)	11 (28)		.47	
	*Giving of self*	6 (15)	9 (23)		.57	
Total Condon test score		22 (55)		21 (53)		>.99

We also compared scores for depressive symptoms between the 2 groups (Table 4). The mean values of the EPDS test and the PDSS test at baseline and follow-up were comparable, and the rates of minor depression, determined by the recommended cutoffs, were also similar. The proportion of lower scores at the follow-up evaluation compared to baseline for the EPDS test was higher in the intervention group than in the control group; however, the difference did not reach statistical significance (53% vs 38%, *P*=.21). The interim questionnaire to evaluate how much the participants recognized the fetal appearance showed an increased proportions of participants with a high score (≥4) in the intervention group. The rate of participants with a high score did not change, or tended to decrease, in the control group (Multimedia Appendix 1, Figure S1).

**Table 4:** Comparison of maternal depressive mood between intervention group and control group.

			Intervention (n=40)		Control (n=40)		*P* value
**Test results at initial baseline evaluation**							
	Gestational age at baseline evaluation (weeks), mean (SD)	27.8 (2.5)		28.2 (3.5)		.498	
	EPDS^a^ score, mean (SD)	6.8 (4.4)		6.1 (3.4)		.43	
	EPDS score ≥10, n (%)	7 (17.5)		5.40 (12.5)		.76	
	PDSS^b^ score, mean (SD)	47.5 (11.4)		48.6 (10.2)		.67	
	PDSS score >60, n (%)	7 (17.5)		5 (12.5)		.76	
**Test results at follow-up evaluation**							
	Gestational age at follow-up evaluation (weeks), mean (SD)	33.3 (2.4)		33.9 (3.0)		.40	
	EPDS score, mean (SD)	6.8 (4.0)		6.3 (2.9)		.57	
	EPDS score ≥10, n (%)	8 (20)		6 (15)		.77	
	Lower EPDS score than initial baseline evaluation, n (%)	21 (52.5)		15 (37.5)		.21	
	PDSS score, mean (SD)	47.5 (7.8)		48.9 (7.8)		.41	
	PDSS score >60, n (%)	3 (7.5)		4 (10)		>.99	
	Lower PDSS score than initial baseline evaluation, n (%)	18 (45)		17 (42.5)		.59	

^a^EPDS: Edinburgh postpartum depression scale.

^b^PDSS: postpartum depression screening scale.

## Discussion

### Principal Findings of the Study

All the participants experienced the prenatal-coaching mobile app, but only the intervention group experienced the VR images. The scores measuring maternal interaction with the fetus showed a significantly greater increase in the intervention group than the control group at follow-up (0.4 vs 0.1). The proportion of participants with increased scores at the follow-up evaluation was over 3 times greater in the intervention group than the control group (43% vs 13%). The intervention group seemed to show improvement in depressive-symptom test scores at the follow-up evaluation, although the difference was not statistically significant (53% vs 38%).

### The Effect of Digital Technologies in Pregnancy

In this study, all participants experienced the mobile prenatal care app, which provided information about what the pregnant women should do and eat. Commercially available apps also provide information on symptoms or signs that pregnant women might feel in each gestational period. The participants were instructed to use the specific prenatal-coaching app that was developed for the trial, but most were already using one or more mobile apps to obtain information about their pregnancy and to record their own diaries or pictures of the fetus. During the pandemic era, the role of digital technology has grown due to the decrease in direct contact, and this has inspired the development of numerous mobile apps for pregnant women to take care of themselves without direct visits to a clinic. Marko et al [48] performed a prospective controlled trial and demonstrated that a mobile prenatal-care app reduced the number of in-person visits without decreasing patient satisfaction. Many prenatal mobile apps contain similar information about routine prenatal tests and currently have additional functions, such as coaching for blood sugar control in patients with gestational diabetes, thereby contributing to improving society by improving the lives of pregnant women and even helping their mental health [49-55].

Taking things further, the digital economy in the postpandemic era has focused on the application of VR to medical fields to produce consistent and easily accessible environments for the management of many disease entities [56,57]. There have been a few intriguing studies involving VR in obstetrics. Wong et al [58] reported that VR was effective in reducing labor pain in a randomized controlled trial. Williams et al [59] demonstrated the application of VR to train midwifery students in emergency skills such as neonatal resuscitation. One pilot study reported that immersive VR reduced anxiety in patients undergoing first-trimester surgical termination [60]. Since ultrasound is the most common tool in obstetrics, VR has been developed based on fetal ultrasounds, and the effectiveness for diagnosing fetal structural anomalies has been tested by several groups [61,62]. Grayscale ultrasound images of the fetus give important information to doctors, but pregnant women and their family members might have difficulty recognizing the face or body parts of the fetus. 3D ultrasounds help people understand the features of the fetus much better than grayscale images. Indeed, when 3D images are combined with VR, the images are far more realistic. Because VR images come from measured ultrasonography data, the fetus can be seen to grow and change in shape at each ultrasonographic examination, and the pregnant woman can easily recognize the differences.

### Clinical Implications

We wanted to determine how fetal images generated in VR can affect maternal-fetal bonding in pregnant women and investigated the possibility that they might lower maternal anxiety or depressive symptoms. This randomized controlled trial showed that there was a significantly greater increase in measured scores of maternal interaction with the fetus in the VR group than the control group. Additionally, although the difference did not reach statistical significance, about half the VR group showed an improvement in depressive-symptom test scores. Cranley [37] defined MFA as “the extent to which women engage in behaviors that represent an affiliation and interaction with their unborn child”; thus, the concept could be considered slightly obscure from a medical perspective. Yarcheski et al [63] performed a meta-analytic study of the predictors of MFA and suggested that gestational age was positively related to increased MFA. Other predicting or associated factors that have been studied are social support for the mother; the mother’s own anxiety, self-esteem, and underlying depression; prenatal test results; whether the pregnancy was planned; maternal age; parity; income; education; and marital status [64-66].

Although MFA could be considered an abstract concept, the association between MFA and postpartum depression has been previously studied. Rollè et al [67] reviewed the literature on the relationship between MFA and perinatal depression and found that lower MFA was related to higher rates of postnatal depressive symptoms, although some of the studies in that review reported controversial or nonsignificant results. Rollè emphasized that the development of strong MFA should be encouraged during pregnancy to reduce postpartum depression and increase the psychological well-being of expectant parents. Modalities to increase healthy and positive MFA should be identified if MFA is to help prevent maternal depressive symptoms, since postpartum depression is a serious psychiatric condition after childbirth that can result in abnormal parenting behavior, negative maternal bonding, child abuse, adverse mental development in childhood, and even maternal suicide [68]. Seimyr et al [69] reported that physical contact with the fetus and sensitivity to fetal movements decreased maternal depression. In fact, an experimental study revealed that watching the fetus in 3D ultrasound images and attempts to touch the fetus in VR decreased maternal stress, as measured by salivary cortisol concentration [70]. Much research has revealed that maternal-fetal bonding increases when pregnant women experience better and more realistic images [71]. Pulliainen et al [72] performed a qualitative pilot study with pregnant women at high risk for preterm birth and showed that interactive 3D ultrasounds shown at the request of the pregnant women increased MFA. A randomized controlled trial of a 4D ultrasound intervention among pregnant women with substance use demonstrated a higher retention rate, as well as enhanced MFA, in the intervention group compared to the control group [73].

### Conclusion

To our knowledge, there are no prior studies demonstrating the effect of VR on maternal-fetal interaction and other aspects of maternal mental status. Fetal images generated in VR seem to promote positive MFA and might reduce maternal depressive symptoms. In this study, we used a prenatal-coaching mobile app to maximize access to fetal images produced with a combination of VR and ultrasound. Since we examined very short-term changes after experiencing VR, future studies are essential to determine the long-term perinatal outcomes of using VR as a modality to increase MFA and to promote mental health during pregnancy.

## Appendix

Multimedia Appendix 1 Contents of questionnaire for maternal-fetal attachment evaluation (Table S1) and proportion of participants with score ≥4 on the questionnaire to assess maternal understanding of fetal appearance (Figure S1).

Multimedia Appendix 2 CONSORT-EHEALTH Checklist V1.6.2.

## Abbreviations

MFA: maternal-fetal attachment
VR: virtual reality
EDPS: Edinburgh Postnatal Depression Scale
PDSS: Postpartum Depression Screening Scale

## References

[1] DeSocio JE (2018). Epigenetics, maternal prenatal psychosocial stress, and infant mental health. Arch Psychiatr Nurs.

[2] Gul F, Sherin A, Jabeen M, Khan SA (2017). Association of stress with anxiety and depression during pregnancy. J Pak Med Assoc.

[3] Reading AE, Cox DN, Sledmere CM, Campbell S (1984). Psychological changes over the course of pregnancy: a study of attitudes toward the fetus/neonate. Health Psychol.

[4] Tomfohr-Madsen LM, Racine N, Giesbrecht GF, Lebel C, Madigan S (2021). Depression and anxiety in pregnancy during COVID-19: A rapid review and meta-analysis. Psychiatry Res.

[5] Hope H, Osam CS, Kontopantelis E, Hughes S, Munford L, Ashcroft DM, Pierce M, Abel KM (2021). The healthcare resource impact of maternal mental illness on children and adolescents: UK retrospective cohort study. Br J Psychiatry.

[6] Field T (2010). Postpartum depression effects on early interactions, parenting, and safety practices: a review. Infant Behav Dev.

[7] Lahti M, Savolainen K, Tuovinen S, Pesonen A, Lahti J, Heinonen K, Hämäläinen Esa, Laivuori H, Villa PM, Reynolds RM, Kajantie E, Räikkönen Katri (2017). Maternal depressive symptoms during and after pregnancy and psychiatric problems in children. J Am Acad Child Adolesc Psychiatry.

[8] Murray L (1992). The impact of postnatal depression on infant development. J Child Psychol Psychiatry.

[9] Murray L, Arteche A, Fearon P, Halligan S, Goodyer I, Cooper P (2011). Maternal postnatal depression and the development of depression in offspring up to 16 years of age. J Am Acad Child Adolesc Psychiatry.

[10] Matthies LM, Müller Mitho, Doster A, Sohn C, Wallwiener M, Reck C, Wallwiener S (2020). Maternal-fetal attachment protects against postpartum anxiety: the mediating role of postpartum bonding and partnership satisfaction. Arch Gynecol Obstet.

[11] Petri E, Palagini L, Bacci O, Borri C, Teristi V, Corezzi C, Faraoni S, Antonelli P, Cargioli C, Banti S, Perugi G, Mauri M (2018). Maternal-foetal attachment independently predicts the quality of maternal-infant bonding and post-partum psychopathology. J Matern Fetal Neonatal Med.

[12] Delavari M, Mohammad-Alizadeh-Charandabi S, Mirghafourvand M (2018). The relationship of maternal-fetal attachment and postpartum depression: a longitudinal study. Arch Psychiatr Nurs.

[13] Arguz Cildir D, Ozbek A, Topuzoglu A, Orcin E, Janbakhishov CE (2020). Association of prenatal attachment and early childhood emotional, behavioral, and developmental characteristics: A longitudinal study. Infant Ment Health J.

[14] Köhler-Dauner Franziska, Buchheim A, Hildebrand K, Mayer I, Clemens V, Ziegenhain U, Fegert JM (2022). Maternal attachment representation, the risk of increased depressive symptoms and the influence on children's mental health during the SARS-CoV-2-pandemic. J Child Fam Stud.

[15] Sacchi C, Miscioscia M, Visentin S, Simonelli A (2021). Maternal-fetal attachment in pregnant Italian women: multidimensional influences and the association with maternal caregiving in the infant's first year of life. BMC Pregnancy Childbirth.

[16] Maddahi MS, Dolatian M, Khoramabadi M, Talebi A (2016). Correlation of maternal-fetal attachment and health practices during pregnancy with neonatal outcomes. Electron Physician.

[17] Zhang L, Wang L, Yuan Q, Huang C, Cui S, Zhang K, Zhou X (2021). The mediating role of prenatal depression in adult attachment and maternal-fetal attachment in primigravida in the third trimester. BMC Pregnancy Childbirth.

[18] Güney Esra, Uçar Tuba (2019). Effect of the fetal movement count on maternal-fetal attachment. Jpn J Nurs Sci.

[19] Chiang D, Huang C, Cheng S, Cheng J, Wu C, Huang S, Yang Ying-Ying, Yang Ling-Yu, Kao Shou-Yen, Chen Chen-Huan, Shulruf Boaz, Lee Fa-Yauh (2022). Immersive virtual reality (VR) training increases the self-efficacy of in-hospital healthcare providers and patient families regarding tracheostomy-related knowledge and care skills: A prospective pre-post study. Medicine (Baltimore).

[20] Venkatesan M, Mohan H, Ryan JR, Schürch Christian M, Nolan GP, Frakes DH, Coskun AF (2021). Virtual and augmented reality for biomedical applications. Cell Rep Med.

[21] Dellazizzo L, Potvin S, Luigi M, Dumais A (2020). Evidence on virtual reality-based therapies for psychiatric disorders: meta-review of meta-analyses. J Med Internet Res.

[22] Kang JM, Kim N, Lee SY, Woo SK, Park G, Yeon BK, Park JW, Youn J, Ryu S, Lee J, Cho S (2021). Effect of cognitive training in fully immersive virtual reality on visuospatial function and frontal-occipital functional connectivity in predementia: randomized controlled trial. J Med Internet Res.

[23] van Loenen I, Scholten W, Muntingh A, Smit J, Batelaan N (2022). The effectiveness of virtual reality exposure-based cognitive behavioral therapy for severe anxiety disorders, obsessive-compulsive disorder, and posttraumatic stress disorder: meta-analysis. J Med Internet Res.

[24] Smith V, Warty RR, Sursas JA, Payne O, Nair A, Krishnan S, da Silva Costa F, Wallace EM, Vollenhoven B (2020). The effectiveness of virtual reality in managing acute pain and anxiety for medical inpatients: systematic review. J Med Internet Res.

[25] Garcia L, Birckhead B, Krishnamurthy P, Mackey I, Sackman J, Salmasi V, Louis R, Castro C, Maddox R, Maddox T, Darnall BD (2022). Durability of the treatment effects of an 8-week self-administered home-based virtual reality program for chronic low back pain: 6-month follow-up study of a randomized clinical trial. J Med Internet Res.

[26] Logan DE, Simons LE, Caruso TJ, Gold JI, Greenleaf W, Griffin A, King CD, Menendez M, Olbrecht VA, Rodriguez S, Silvia M, Stinson JN, Wang E, Williams SE, Wilson L (2021). Leveraging virtual reality and augmented reality to combat chronic pain in youth: position paper from the interdisciplinary network on virtual and augmented technologies for pain management. J Med Internet Res.

[27] Lungu AJ, Swinkels W, Claesen L, Tu P, Egger J, Chen X (2021). A review on the applications of virtual reality, augmented reality and mixed reality in surgical simulation: an extension to different kinds of surgery. Expert Rev Med Devices.

[28] Sanford DI, Ma R, Ghoreifi A, Haque TF, Nguyen JH, Hung AJ (2022). Association of suturing technical skill assessment scores between virtual reality simulation and live surgery. J Endourol.

[29] Chen J, Or CK, Chen T (2022). Effectiveness of using virtual reality-supported exercise therapy for upper extremity motor rehabilitation in patients with stroke: systematic review and meta-analysis of randomized controlled trials. J Med Internet Res.

[30] Chen F, Leng Y, Ge J, Wang D, Li C, Chen B, Sun Z (2020). Effectiveness of virtual reality in nursing education: meta-analysis. J Med Internet Res.

[31] Perron JE, Coffey MJ, Lovell-Simons A, Dominguez L, King ME, Ooi CY (2021). Resuscitating cardiopulmonary resuscitation training in a virtual reality: prospective interventional study. J Med Internet Res.

[32] Sjögren B, Edman G, Widström AM, Mathiesen AS, Uvnäs‐Moberg K (2004). Maternal foetal attachment and personality during first pregnancy. J Reprod Infant Psychol.

[33] Kang Hyun (2021). Sample size determination and power analysis using the G*Power software. J Educ Eval Health Prof.

[34] Doster A, Wallwiener S, Müller Mitho, Matthies LM, Plewniok K, Feller S, Kuon R, Sohn C, Rom J, Wallwiener M, Reck C (2018). Reliability and validity of the German version of the Maternal-Fetal Attachment Scale. Arch Gynecol Obstet.

[35] Müller M E, Ferketich S (1993). Factor analysis of the Maternal Fetal Attachment Scale. Nurs Res.

[36] Pohárnok Melinda, Kopcsó Krisztina, Polgár Petra Ibolya (2022). The structure and correlates of the 20-item Maternal-Fetal Attachment Scale in a population-based sample of Hungarian expectant women. Midwifery.

[37] Cranley MS (1981). Development of a tool for the measurement of maternal attachment during pregnancy. Nurs Res.

[38] Lingeswaran A, Bindu H (2012). Validation of Tamil version of Cranley's 24-item Maternal-Fetal Attachment Scale in Indian pregnant women. J Obstet Gynaecol India.

[39] Condon J (1993). The assessment of antenatal emotional attachment: development of a questionnaire instrument. Br J Med Psychol.

[40] Tanuma-Takahashi A, Tanemoto T, Nagata C, Yokomizo R, Konishi A, Takehara K, Ishikawa T, Yanaihara N, Samura O, Okamoto A (2022). Antenatal screening timeline and cutoff scores of the Edinburgh Postnatal Depression Scale for predicting postpartum depressive symptoms in healthy women: a prospective cohort study. BMC Pregnancy Childbirth.

[41] O'Connor E, Rossom RC, Henninger M, Groom HC, Burda BU (2016). Primary care screening for and treatment of depression in pregnant and postpartum women: evidence report and systematic review for the US Preventive Services Task Force. JAMA.

[42] Cox JL, Holden JM, Sagovsky R (1987). Detection of postnatal depression. Development of the 10-item Edinburgh Postnatal Depression Scale. Br J Psychiatry.

[43] Park S, Kim J (2022). Predictive validity of the Edinburgh postnatal depression scale and other tools for screening depression in pregnant and postpartum women: a systematic review and meta-analysis. Arch Gynecol Obstet.

[44] Horáková Anna, Nosková Eliška, Švancer Patrik, Marciánová Vladislava, Koliba P, Šebela Antonín (2022). Accuracy of the Edinburgh Postnatal Depression Scale in screening for major depressive disorder and other psychiatric disorders in women towards the end of their puerperium. Ceska Gynekol.

[45] Mao F, Sun Y, Wang J, Huang Y, Lu Y, Cao F (2021). Sensitivity to change and minimal clinically important difference of Edinburgh postnatal depression scale. Asian J Psychiatr.

[46] Beck CT, Gable RK (2000). Postpartum Depression Screening Scale: development and psychometric testing. Nurs Res.

[47] Beck CT, Gable RK (2001). Comparative analysis of the performance of the Postpartum Depression Screening Scale with two other depression instruments. Nurs Res.

[48] Marko KI, Ganju N, Krapf JM, Gaba ND, Brown JA, Benham JJ, Oh J, Richards LM, Meltzer AC (2019). A mobile prenatal care app to reduce in-person visits: prospective controlled trial. JMIR Mhealth Uhealth.

[49] Garnweidner-Holme L, Henriksen L, Torheim LE, Lukasse M (2020). Effect of the Pregnant+ smartphone app on the dietary behavior of women with gestational diabetes mellitus: secondary analysis of a randomized controlled trial. JMIR Mhealth Uhealth.

[50] Skar JB, Garnweidner-Holme LM, Lukasse M, Terragni L (2018). Women's experiences with using a smartphone app (the Pregnant+ app) to manage gestational diabetes mellitus in a randomised controlled trial. Midwifery.

[51] Souza FMDLC, Santos WND, Santos RSDC, Silva VLMD, Abrantes RMD, Soares VFR, Silva RARD (2021). Effectiveness of mobile applications in pregnant women's adherence to prenatal consultations: randomized clinical trial. Rev Bras Enferm.

[52] Wu K, Alegria R, Gonzalez J, Hu H, Wang H, Page R, Robbins-Furman P, Ma P, Tseng T, Chen L (2021). Characteristics and quality of mobile apps containing prenatal genetic testing information: systematic app store search and assessment. JMIR Mhealth Uhealth.

[53] Smith R, Mahnert N, Foote J, Saunders K, Mourad J, Huberty J (2021). Mindfulness effects in obstetric and gynecology patients during the Coronavirus Disease 2019 (COVID-19) pandemic: a randomized controlled trial. Obstet Gynecol.

[54] Saad A, Magwood O, Aubry T, Alkhateeb Q, Hashmi SS, Hakim J, Ford L, Kassam A, Tugwell P, Pottie K (2021). Mobile interventions targeting common mental disorders among pregnant and postpartum women: An equity-focused systematic review. PLoS One.

[55] Bogaerts A, Bijlholt M, Mertens L, Braeken M, Jacobs B, Vandenberghe B, Ameye L, Devlieger R (2020). Development and Field Evaluation of the INTER-ACT App, a Pregnancy and Interpregnancy Coaching App to Reduce Maternal Overweight and Obesity: Mixed Methods Design. JMIR Form Res.

[56] Vlake JH, van Bommel J, Wils E, Bienvenu J, Hellemons ME, Korevaar TI, Schut AF, Labout JA, Schreuder LL, van Bavel MP, Gommers D, van Genderen ME (2022). Intensive Care Unit-Specific Virtual Reality for Critically Ill Patients With COVID-19: Multicenter Randomized Controlled Trial. J Med Internet Res.

[57] Zhang Q, Fu Y, Lu Y, Zhang Y, Huang Q, Yang Y, Zhang K, Li M (2021). Impact of Virtual Reality-Based Therapies on Cognition and Mental Health of Stroke Patients: Systematic Review and Meta-analysis. J Med Internet Res.

[58] Wong MS, Spiegel BM, Gregory KD (2021). Virtual Reality Reduces Pain in Laboring Women: A Randomized Controlled Trial. Am J Perinatol.

[59] Williams J, Jones D, Walker R (2018). Consideration of using virtual reality for teaching neonatal resuscitation to midwifery students. Nurse Educ Pract.

[60] Sridhar A, Shiliang Z, Woodson R, Kwan L (2020). Non-pharmacological anxiety reduction with immersive virtual reality for first-trimester dilation and curettage: a pilot study. Eur J Contracept Reprod Health Care.

[61] Pietersma CS, Mulders AGMGJ, Moolenaar LM, Hunink MGM, Koning AHJ, Willemsen SP, Go ATJI, Steegers EAP, Rousian M (2020). First trimester anomaly scan using virtual reality (VR FETUS study): study protocol for a randomized clinical trial. BMC Pregnancy Childbirth.

[62] Werner H, Lopes Dos Santos JR, Ribeiro G, Belmonte SL, Daltro P, Araujo Júnior E (2017). Combination of ultrasound, magnetic resonance imaging and virtual reality technologies to generate immersive three-dimensional fetal images. Ultrasound Obstet Gynecol.

[63] Yarcheski A, Mahon NE, Yarcheski TJ, Hanks MM, Cannella BL (2009). A meta-analytic study of predictors of maternal-fetal attachment. Int J Nurs Stud.

[64] Muller M E (1992). A critical review of prenatal attachment research. Sch Inq Nurs Pract.

[65] Stroup DF, Berlin J A, Morton S C, Olkin I, Williamson G D, Rennie D, Moher D, Becker B J, Sipe T A, Thacker S B (2000). Meta-analysis of observational studies in epidemiology: a proposal for reporting. Meta-analysis Of Observational Studies in Epidemiology (MOOSE) group. JAMA.

[66] Rees BL (1980). Measuring identification with the mothering role. Res Nurs Health.

[67] Rollè Luca, Giordano M, Santoniccolo F, Trombetta T (2020). Prenatal Attachment and Perinatal Depression: A Systematic Review. Int J Environ Res Public Health.

[68] Zhao X, Zhang Z (2020). Risk factors for postpartum depression: An evidence-based systematic review of systematic reviews and meta-analyses. Asian J Psychiatr.

[69] Seimyr L, Sjögren Berit, Welles-Nyström Barbara, Nissen E (2009). Antenatal maternal depressive mood and parental-fetal attachment at the end of pregnancy. Arch Womens Ment Health.

[70] Severi F M, Prattichizzo D, Casarosa E, Barbagli F, Ferretti C, Altomare A, Vicino A, Petraglia F (2005). Virtual fetal touch through a haptic interface decreases maternal anxiety and salivary cortisol. J Soc Gynecol Investig.

[71] de Jong-Pleij E A P, Ribbert LSM, Pistorius LR, Tromp E, Mulder EJH, Bilardo CM (2013). Three-dimensional ultrasound and maternal bonding, a third trimester study and a review. Prenat Diagn.

[72] Pulliainen H, Niela-Vilén Hannakaisa, Ekholm E, Ahlqvist-Björkroth Sari (2019). Experiences of interactive ultrasound examination among women at risk of preterm birth: a qualitative study. BMC Pregnancy Childbirth.

[73] Jussila H, Pajulo M, Ekholm E (2020). A Novel 4D Ultrasound Parenting Intervention for Substance Using Pregnant Women in Finland: Participation in Obstetric Care, Fetal Drug Exposure, and Perinatal Outcomes in a Randomized Controlled Trial. Matern Child Health J.

