# Supplementary material for: Effects of Fetal Images Produced in Virtual Reality on Maternal-Fetal Attachment: Randomized Controlled Trial
Source: J Med Internet Res. 2023 Feb 24;25:e43634. doi: 10.2196/43634 (PMC10007014; doi:10.2196/43634)
Supplement: Multimedia Appendix 1 [file jmir_v25i1e43634_app1.docx]

**Supplementary table 1.** Contents of questionnaire for maternal-fetal attachment evaluation

| **Question** | **Category** | **Answer** | |
| --- | --- | --- | --- |
| **Cranley test** | | |  |
| 1. I talk to my unborn child | interaction with the fetus | - Choices:  1. Definitely No  2. No  3. Uncertain  4. Yes  5. Definitely Yes  - Negative item number 22 was scored reversely  - The total score ranged from 24 to 120 | |
| 1. I feel all the trouble of being pregnant is worth it | giving of self |  |  |
| 1. I enjoy watching my tummy jiggle as the baby kicks inside | differentiation of self from fetus |  |  |
| 1. I picture myself feeding the baby | role taking |  |  |
| 1. I'm really looking forward to seeing what the baby looks like | differentiation of self from fetus |  |  |
| 1. I wonder if the baby feels cramped in there | attributing characteristics to fetus |  |  |
| 1. I refer to my baby by a nickname | interaction with the fetus |  |  |
| 1. I imagine myself taking care of the baby | role taking |  |  |
| 1. I can almost guess what my baby's personality will be like from the way she/he moves around | attributing characteristics to fetus |  |  |
| 1. If my baby is a girl, I have chosen a name for her already | differentiation of self from fetus |  |  |
| 1. I do things to try to stay healthy that I would not do if I were not pregnant | giving of self |  |  |
| 1. I wonder if the baby can hear inside of me | attributing characteristics to fetus |  |  |
| 1. I have decided on a name for a baby boy | differentiation of self from fetus |  |  |
| 1. I wonder if the baby thinks and feels inside of me | attributing characteristics to fetus |  |  |
| 1. I eat meat and vegetables to be sure my baby gets a good diet | giving of self |  |  |
| 1. It seems the baby kicks and moves just to remind me that it is my feeding time | attributing characteristics to fetus |  |  |
| 1. I poke the baby to get him/her to poke back | interaction with the fetus |  |  |
| 1. I can hardly wait to hold the baby | role taking |  |  |
| 1. I try to picture what the baby will look like | role taking |  |  |
| 1. I stroke my tummy to quiet the baby when there is too much kicking | interaction with the fetus |  |  |
| 1. I can tell the baby has hiccoughs | attributing characteristics to fetus |  |  |
| 1. I feel my body is ugly | giving of self |  |  |
| 1. I give up doing certain things because I want to help my baby | giving of self |  |  |
| 1. I grasp my baby's foot through my tummy to move it around | interaction with the fetus |  |  |
| **Codon test** | | |  |
| 1. Frequent/infrequent picturing fetus in imagination |  | - From negative to positive answer, the choice of answers was given from 1 to 5  - The total score ranged from 19 to 95 | |
| 1. Strong/weak feelings accompanying thoughts of fetus |  |  |  |
| 1. Positive/negative feelings towards fetus |  |  |  |
| 1. Strong/weak desire to read or get information about fetus |  |  |  |
| 1. Frequent/infrequent picturing fetus in imagination |  |  |  |
| 1. Concept of fetus as "person"/"thing" |  |  |  |
| 1. Fetus dependent for well-being |  |  |  |
| 1. Frequent/infrequent talking to fetus |  |  |  |
| 1. Tender/irritable feelings towards fetus |  |  |  |
| 1. Clear/vague mental picture of fetus |  |  |  |
| 1. Happy/sad feelings about fetus |  |  |  |
| 1. Absence/presence of desire to hurt or punish fetus |  |  |  |
| 1. Feeling emotionally close to/distant from fetus |  |  |  |
| 1. Frequent/infrequent concern on mother's diet |  |  |  |
| 1. Anticipate positive/negative first impression of baby |  |  |  |
| 1. Desire to hold baby immediately/later |  |  |  |
| 1. Frequent/infrequent dreams about baby |  |  |  |
| 1. Frequent/infrequent palpation of fetus |  |  |  |
| 1. Sadness/mixed feelings towards fantasized fetal loss |  |  |  |


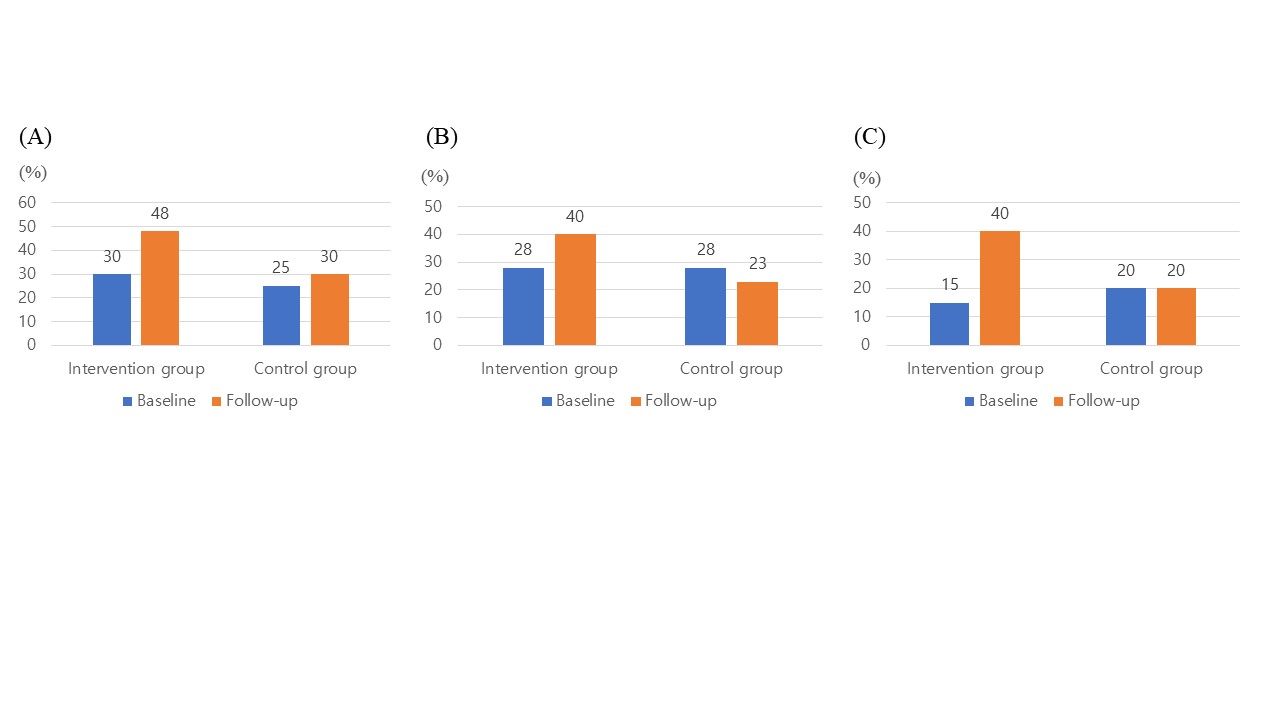


**Supplementary Figure.** Proportions of participants with score ≥ 4 at the questionaire to assess maternal understanding for fetal appearance (the score ranged from 1 to 5; 1, definitely no; 2, very little; 3, moderately; 4, very much; 5, definitely yes). The questions were as follows: (A) Do you understand and imagine how big the actual size of your baby is? ; (B) Do you understand and imagine the lengths of your baby’s arms and legs?; (C) Do you understand and imagine the detailed appearance of your baby’s face?
