# Supplementary material for: Effects of Fetal Images Produced in Virtual Reality on Maternal-Fetal Attachment: Randomized Controlled Trial
Source: J Med Internet Res. 2023 Feb 24;25:e43634. doi: 10.2196/43634 (PMC10007014; doi:10.2196/43634)
Supplement: Multimedia Appendix 2 [file jmir_v25i1e43634_app2.pdf]

CONSORT-EHEALTH Checklist V1.6.2 Report	Manuscript Number	43634

(based on CONSORT-EHEALTH V1.6), available at [http://tinyurl.com/consort-ehealth-v1-6].

Date completed

1/26/2023 1:10:45

by

Kyong-No Lee

Effects of Fetal Images Produced by Virtual Reality on Maternal-fetal Attachment: Randomized Clinical Trial

TITLE

1a-i) Identify the mode of delivery in the title

"Effects of Fetal Images Produced by Virtual Reality on Maternal-fetal Attachment: Randomized Clinical Trial"

1a-ii) Non-web-based components or important co-interventions in title

1a-iii) Primary condition or target group in the title

"Effects of Fetal Images Produced by Virtual Reality on Maternal-fetal Attachment: Randomized Clinical Trial"

ABSTRACT

1b-i) Key features/functionalities/components of the intervention and comparator in the METHODS section of the ABSTRACT

"Eligible women who provided written informed consent were randomly assigned to either the mobile app only group (n=40) or the app with VR group (n=40)."

1b-ii) Level of human involvement in the METHODS section of the ABSTRACT

1b-iii) Open vs. closed, web-based (self-assessment) vs. face-to-face assessments in the METHODS section of the ABSTRACT

1b-iv) RESULTS section in abstract must contain use data

1b-v) CONCLUSIONS/DISCUSSION in abstract for negative trials

INTRODUCTION

2a-i) Problem and the type of system/solution

"The aim was to determine the effect of fetal images generated by VR to MFA and depressive symptoms through prenatal coaching mobile app."

2a-ii) Scientific background, rationale: What is known about the (type of) system

"maternal strong emotion toward bonding to her baby had protective effects on postpartum depression or anxiety and tried to find out the factors to stimulate or improve maternal-fetal attachment"

"Virtual reality (VR) is the newest trend in computer-based technology that stimulates the recognition of certain objects or situation realistically regardless of location or time. It has been applied to numerous fields of health care such as providing treatment, facilitating pain management, surgery simulation, guidance for rehabilitation, and medical education."

Does your paper address CONSORT subitem 2b?

"Since VR can reproduce far more real appearance of fetus with the assistance of ultrasonography, we hypothesized the vivid images of fetus through VR could help promote MFA."

METHODS

3a) CONSORT: Description of trial design (such as parallel, factorial) including allocation ratio

"The 2-arm parallel randomized controlled trial was conducted at the Department of Obstetrics and Gynecology, the Seoul National University Bundang Hospital, Republic of Korea."

3b) CONSORT: Important changes to methods after trial commencement (such as eligibility criteria), with reasons

"Participants were recruited among the pregnant women who visited the institution for routine prenatal check-ups and pregnancies after 20 weeks of gestation were identified to be eligible. Patients with a history of any psychiatric disorders (e.g., mood disorder or anxiety disorder) were initially excluded from this study."

3b-i) Bug fixes, Downtimes, Content Changes

4a) CONSORT: Eligibility criteria for participants

"Participants were recruited among the pregnant women who visited the institution for routine prenatal check-ups and pregnancies after 20 weeks of gestation were identified to be eligible. Patients with a history of any psychiatric disorders (e.g., mood disorder or anxiety disorder) were initially excluded from this study."

4a-i) Computer / Internet literacy

4a-ii) Open vs. closed, web-based vs. face-to-face assessments:

"Every questionnaire was provided to the participants before and after fetal ultrasound examination at the outpatient clinic and the average time for completion of each questionnaire was approximately 10 minutes."

4a-iii) Information giving during recruitment

4b) CONSORT: Settings and locations where the data were collected

"The 2-arm parallel randomized controlled trial was conducted at the Department of Obstetrics and Gynecology, the Seoul National University Bundang Hospital, Republic of Korea."

4b-i) Report if outcomes were (self-)assessed through online questionnaires

"All participants completed the trial in 4 weeks from the enrollment and at the end of trial, they repeated the three types of questionnaires that they had done at the beginning of the study (Figure 1). Every questionnaire was provided to the participants before and after fetal ultrasound examination at the outpatient clinic and the average time for completion of each questionnaire was approximately 10 minutes."

4b-ii) Report how institutional affiliations are displayed

5) CONSORT: Describe the interventions for each group with sufficient details to allow replication, including how and when they were actually administered

5-i) Mention names, credential, affiliations of the developers, sponsors, and owners

5-ii) Describe the history/development process

5-iii) Revisions and updating

5-iv) Quality assurance methods

5-v) Ensure replicability by publishing the source code, and/or providing screenshots/screen-capture video, and/or providing flowcharts of the algorithms used

5-vi) Digital preservation

5-vii) Access

"All participants received the prenatal coaching mobile app called Aluvuu app (both Android and iOS compatible, Girjae Soft CO., Ltd., Republic of Korea) as they were enrolled."

5-viii) Mode of delivery, features/functionalities/components of the intervention and comparator, and the theoretical framework

", all participants underwent three-dimensional (3D) fetal ultrasonography and could see the fetal images on ultrasonography monitor, however only the VR intervention group additionally experienced fetal images merged from ultrasonographic data to VR simulator after wearing the headset. The fetal images were generated by VR from the information gained through each ultrasonography of the subject’s own fetus and were given only to the VR group."

5-ix) Describe use parameters

5-x) Clarify the level of human involvement

5-xi) Report any prompts/reminders used

"Prenatal coaching mobile app was provided afterwards and the consistent education to use the various functions of the app was given to all participants. Approximately two weeks later, the researchers checked the interim feedback for the proper use of prenatal coaching mobile app and the fourth questionnaire to assess maternal understanding of fetal appearance was given to all participants."		
**5-xii) Describe any co-interventions (incl. training/support)**		
"Prenatal coaching mobile app was provided afterwards and the consistent education to use the various functions of the app was given to all participants. Approximately two weeks later, the researchers checked the interim feedback for the proper use of prenatal coaching mobile app and the fourth questionnaire to assess maternal understanding of fetal appearance was given to all participants."		
**6a) CONSORT: Completely defined pre-specified primary and secondary outcome measures, including how and when they were assessed**		
"The major questionnaire was to assess MFA, which is the primary outcome of this study. Two types of well-known evaluation scoring systems previously published were used: Cranley method and Condon method (Supplementary table)."		
**6a-i) Online questionnaires: describe if they were validated for online use and apply CHERRIES items to describe how the questionnaires were designed/deployed**		
**6a-ii) Describe whether and how "use" (including intensity of use/dosage) was defined/measured/monitored**		
**6a-iii) Describe whether, how, and when qualitative feedback from participants was obtained**		
**6b) CONSORT: Any changes to trial outcomes after the trial commenced, with reasons**		
"The 2-arm parallel randomized controlled trial was conducted at the Department of Obstetrics and Gynecology, the Seoul National University Bundang Hospital, Republic of Korea."		
**7a) CONSORT: How sample size was determined**		
**7a-i) Describe whether and how expected attrition was taken into account when calculating the sample size**		
**7b) CONSORT: When applicable, explanation of any interim analyses and stopping guidelines**		
"The major questionnaire was to assess MFA, which is the primary outcome of this study. Two types of well-known evaluation scoring systems previously published were used: Cranley method and Condon method (Supplementary table)."		
**8a) CONSORT: Method used to generate the random allocation sequence**		
"During the study period, 88 eligible women were recruited and assigned randomly to either the VR intervention group or standard care group in a ratio of 1: 1 by restricted randomization generated from statistical software by the Medical Research Collaborating Center of the institution."		
**8b) CONSORT: Type of randomisation; details of any restriction (such as blocking and block size)**		
"The 2-arm parallel randomized controlled trial"		
**9) CONSORT: Mechanism used to implement the random allocation sequence (such as sequentially numbered containers), describing any steps taken to conceal the sequence until interventions were assigned**		
"During the study period, 88 eligible women were recruited and assigned randomly to either the VR intervention group or standard care group in a ratio of 1: 1 by restricted randomization generated from statistical software by the Medical Research Collaborating Center of the institution. Clinicians who met the participants in practice were not involved in randomization process and were blinded to the allocation in each group until the participants completed all the study protocol."		
**10) CONSORT: Who generated the random allocation sequence, who enrolled participants, and who assigned participants to interventions**		
The researcher in charge of the study conducted.		
**11a) CONSORT: Blinding - If done, who was blinded after assignment to interventions (for example, participants, care providers, those assessing outcomes) and how**		
**11a-i) Specify who was blinded, and who wasn't**		
"During the study period, 88 eligible women were recruited and assigned randomly to either the VR intervention group or standard care group in a ratio of 1: 1 by restricted randomization generated from statistical software by the Medical Research Collaborating Center of the institution (Figure 1). Clinicians who met the participants in practice were not involved in randomization process and were blinded to the allocation in each group until the participants completed all the study protocol."		
**11a-ii) Discuss e.g., whether participants knew which intervention was the "intervention of interest" and which one was the "comparator"**		
**11b) CONSORT: If relevant, description of the similarity of interventions**		
"all participants underwent three-dimensional (3D) fetal ultrasonography and could see the fetal images on ultrasonography monitor, however only the VR intervention group additionally experienced fetal images merged from ultrasonographic data to VR simulator after wearing the headset."		
**12a) CONSORT: Statistical methods used to compare groups for primary and secondary outcomes**		
"Maternal obstetric information and the scores from all the questionnaires were compared according to the groups. Continuous variables were analyzed by Student's t-test and proportions were compared using Fisher's exact test. A p-value of < 0.05 was considered significant. All statistical analyses were performed using IBM SPSS Statistics, version 25.0"		
**12a-i) Imputation techniques to deal with attrition / missing values**		
"Among 88 participants enrolled at the beginning, four from each group were lost to follow up, therefore the data from 80 participants were included and analyzed finally."		
**12b) CONSORT: Methods for additional analyses, such as subgroup analyses and adjusted analyses**		
"The scores measured to assess depressive symptoms were analyzed between two groups."		
RESULTS		
**13a) CONSORT: For each group, the numbers of participants who were randomly assigned, received intended treatment, and were analysed for the primary outcome**		
"After they finished the three questionnaires, all participants underwent three-dimensional (3D) fetal ultrasonography and could see the fetal images on ultrasonography monitor, however only the VR intervention group additionally experienced fetal images merged from ultrasonographic data to VR simulator after wearing the headset. The fetal images were generated by VR from the information gained through each ultrasonography of the subject's own fetus and were given only to the VR group. Prenatal coaching mobile app was provided afterwards and the consistent education to use the various functions of the app was given to all participants."		
**13b) CONSORT: For each group, losses and exclusions after randomisation, together with reasons**		
"Among 88 participants enrolled at the beginning, four from each group were lost to follow up, therefore the data from 80 participants were included and analyzed finally."		
**13b-i) Attrition diagram**		
**14a) CONSORT: Dates defining the periods of recruitment and follow-up**		
"Prior to initiation of the study, the trial was approved by the Seoul National University Bundang Hospital Institutional Review Board (B-2106-688-302) and the protocol was registered to clinicaltrials.gov (NCT04942197). Written informed consent was obtained from all participants. Recruitment began in June 2021 and enrollment was completed in October 2021." "All participants completed the trial in 4 weeks from the enrollment and at the end of trial, they repeated the three types of questionnaires that they had done at the beginning of the study."		
**14a-i) Indicate if critical "secular events" fell into the study period**		
**14b) CONSORT: Why the trial ended or was stopped (early)**		
We enroll as much as the target population of this study. And the study was ended.		
**15) CONSORT: A table showing baseline demographic and clinical characteristics for each group**		
Table 1 shows baseline demographics and clinical characteristics of the participants		
**15-i) Report demographics associated with digital divide issues**		
Table 1 shows the age and education level		
**16a) CONSORT: For each group, number of participants (denominator) included in each analysis and whether the analysis was by original assigned groups**		
**16-i) Report multiple "denominators" and provide definitions**		
"The target sample size was calculated based on the change of Cranley scores from the previous study. Given the average Cranley attachment score of 2.8 (standard deviation 0.51), thirty-two patients were required in each study arm to determine an increase in Cranley score of 0.3 using the software G*Power 3 with an α value of 0.05, a power of 80% (β = 0.20), and a two-tailed test. Allowing for about 25% dropout rate, we decided to enroll a total of 88 patients (44 participants per each group)."		
**16-ii) Primary analysis should be intent-to-treat**		
**17a) CONSORT: For each primary and secondary outcome, results for each group, and the estimated effect size and its precision (such as 95% confidence interval)**		

"The total scores tested by Cranley method and Codon method did not show statistical difference, both at the baseline evaluation and at the follow-up, however looking into the change from the beginning to the follow-up, the mean value of one subscale in Cranley test, interaction with the fetus, increased higher in the intervention group than in the control group (0.4±0.5 vs. 0.1±0.4, p=0.004). Figure 5 reveals the proportions of participants who had increased scores at follow-up evaluation compared to initial baseline results. The rate of participants with change to higher score was 43% for the intervention group while it was 13% for the control group (p=0.005)."		
**17a-i) Presentation of process outcomes such as metrics of use and intensity of use**		
**17b) CONSORT: For binary outcomes, presentation of both absolute and relative effect sizes is recommended**		
Since it is a score measurement through questionnaire, it is not a binary outcome.		
**18) CONSORT: Results of any other analyses performed, including subgroup analyses and adjusted analyses, distinguishing pre-specified from exploratory**		
The analysis results are included in Tables 1 through 4.		
**18-i) Subgroup analysis of comparing only users**		
**19) CONSORT: All important harms or unintended effects in each group**		
At the time of the IRB review, it was said that mothers wearing VR could complain of nausea or dizziness. In the case of mothers who complained, all recovered after a short rest.		
**19-i) Include privacy breaches, technical problems**		
**19-ii) Include qualitative feedback from participants or observations from staff/researchers**		
DISCUSSION		
**20) CONSORT: Trial limitations, addressing sources of potential bias, imprecision, multiplicity of analyses**		
**20-i) Typical limitations in ehealth trials**		
This study has nothing to do with ehealth trial limitations.		
**21) CONSORT: Generalisability (external validity, applicability) of the trial findings**		
**21-i) Generalizability to other populations**		
**21-ii) Discuss if there were elements in the RCT that would be different in a routine application setting**		
**22) CONSORT: Interpretation consistent with results, balancing benefits and harms, and considering other relevant evidence**		
**22-i) Restate study questions and summarize the answers suggested by the data, starting with primary outcomes and process outcomes (use)**		
"All the participants experienced the prenatal coaching mobile app, but the VR was applied only to the intervention group. The scores measuring maternal interaction with the fetus significantly increased higher in the intervention group than in the control group at the follow-up tests (0.4 vs. 0.1). The proportion of participants with increased scores at the follow-up evaluation was over three times for the intervention group compared to that of the control group (43% vs. 13%). The intervention group seemed to improve the depressive symptom test scores at the follow-up evaluation although the difference was not statistically significant (53% vs. 38%)."		
**22-ii) Highlight unanswered new questions, suggest future research**		
Other information		
**23) CONSORT: Registration number and name of trial registry**		
"clinicaltrials.gov (NCT04942197)"		
**24) CONSORT: Where the full trial protocol can be accessed, if available**		
"the protocol was registered to clinicaltrials.gov (NCT04942197)."		
**25) CONSORT: Sources of funding and other support (such as supply of drugs), role of funders**		
"This work was funded by the National Research Foundation of Korea (NRF), the Ministry of Small and Medium-sized Enterprises and Startups (Grant project number S2798153). Girjae Soft CO., Ltd. was supported by this fund and developed the mobile app and virtual reality technology for fetal images. The researchers are independent of the funding bodies."		
**X26-i) Comment on ethics committee approval**		
**x26-ii) Outline informed consent procedures**		
**X26-iii) Safety and security procedures**		
**X27-i) State the relation of the study team towards the system being evaluated**		
